# Evaluation of the Characteristics and Machine Learning-Based Identification Accuracy of the International Electrotechnical Commission (IEC) 60601-1-8 Medical Equipment Auditory Alarms

**DOI:** 10.7759/cureus.82488

**Published:** 2025-04-18

**Authors:** Kai Ishida, Kiyotaka Fujii

**Affiliations:** 1 Faculty of Engineering, Shonan Institute of Technology, Fujisawa, JPN; 2 School of Allied Health Sciences, Kitasato University, Minato, JPN

**Keywords:** auditory alarm, cosine similarity, machine learning, medical equipment, random forest, support vector machine

## Abstract

Introduction

Auditory alarms in clinical settings signal sudden changes in a patient’s condition and failures in medical equipment. However, distinguishing between simultaneously sounding alarms, particularly when superimposed with various ambient sounds, remains challenging. This study aimed to develop a machine learning (ML) model for identifying auditory alarms issued by medical equipment.

Methods

We targeted old and new auditory alarms for medical equipment as specified in the International Electrotechnical Commission (IEC) 60601-1-8 standard. First, we evaluated the characteristics of both normal and degraded auditory alarms using cosine similarity among old and new alarms. Next, we evaluated the accuracy of ML-based identification of deteriorated alarm sound sources in both the old and new alarm groups.

Results

The cosine similarity among old alarms was over 0.99, while new alarms ranged from 0.886 to 0.985, and exhibited more distinct characteristics. When noise was superimposed, the similarity among old alarms increased further, making differentiation more difficult. In contrast, for most new alarms, cosine similarity values exceeded 0.99 but retained slight acoustic differences even after noise-induced degradation, demonstrating improved distinguishability. The accuracy for identifying a single degraded alarm sound was 71.9% for the support vector machine. The models exhibited a high number of misclassifications when identifying the old alarms. Conversely, the models achieved higher accuracy when classifying new alarms, with recall exceeding 80%, precision above 70%, and F-measure greater than 80% for all new alarms. The identification accuracies for two simultaneous alarms were under 20% and approximately 50% for old and new alarms, respectively. The accuracy declined when estimating two simultaneous new alarms; however, when at least one of the two alarms was correctly classified, the accuracy exceeded 90%.

Conclusions

This study evaluated the characteristics of old and new auditory alarms issued by medical equipment as specified in IEC 60601-1-8 and constructed ML models for identifying the type of alarms.

## Introduction

Medical equipment and systems are equipped with auditory alarm systems to alert medical personnel about abrupt changes in patient status, operational abnormalities, or malfunctions. However, questionnaire survey data on the efficiency of medical equipment auditory alarms indicate instances of missed alarms and confusion with other auditory signals [1]. For instance, a 2017 survey revealed that approximately 40% of medical incidents and near-misses caused by problems related to patient monitor and equipment alarms occurred despite the triggering of alarm signals [2]. According to previous research, ambient noise sources in intensive care units include staff conversations, door opening and closing, footsteps, operating equipment, and medical equipment alarms [3]. In medical environments, particularly in intensive care and critical care units, auditory alarms sound frequently. Although lacking a standardized definition, alarm fatigue, wherein staff become desensitized owing to excessive alarm exposure, is considered a major concern [4]. Consequently, many hospitals are implementing alarm reduction strategies [5-8]. For instance, technical alarms indicating mechanical issues, such as electrode or probe disconnections and signal loss, can be minimized through thorough daily equipment checks [8]. However, reducing the frequency of high-priority alarms indicating physiological abnormalities or sudden changes in patient vital signs remains challenging, emphasizing the importance of reliable alarm recognition and response measures.

The International Electrotechnical Commission (IEC) 60601-1-8 standard specifies general requirements for medical equipment alarm systems [9]. Revisions to this standard in 2020 introduced changes to the alarm categorization criteria outlined in the 2012 version. Additionally, the tonal characteristics assigned to each alarm category were significantly modified to enhance ergonomic effectiveness. This revision was prompted by findings indicating that the pulse alarm tone specified in the previous edition was difficult to memorize and prone to misinterpretation [10]. The auditory alarm tones defined in the revised standard have been reported to enable improved auditory discrimination and detectability compared to previous versions [11]. However, humans are continuously exposed to environmental sounds, which impose inherent limitations on the accurate recognition of simultaneous alarms.

In addition to auditory alarms, supplementary visual alert systems, such as flashing or illuminated indicators, are also used in medical settings. However, these systems present inherent limitations. An alternative approach involves networking medical devices within a facility and integrating them with the hospital information system (HIS). For instance, patient monitors, infusion pumps, and ventilators can be connected to the HIS through a wireless local area network, enabling continuous monitoring of their operational status. In this scenario, system malfunctions trigger appropriate alarms [12,13]. However, the implementation of such systems encounters several challenges: communication protocol differences between medical equipment and HIS manufacturers complicate system construction, many medical equipment systems lack external output terminals, system construction costs remain high, and collaboration between stakeholders remains limited. Alternatively, recent research has explored the application of sound recognition and machine learning (ML) using edge devices [14]. ML has the potential to process complex acoustic patterns, improve detection in noisy environments, and reduce misclassification. For instance, if an edge device can detect auditory alarms and extract alarm information, it may enable seamless HIS integration for equipment lacking external output terminals. However, no studies have yet examined the identification accuracy of both the old and new alarm tones specified in IEC 60601-1-8 using ML approaches. Moreover, actual clinical environments present additional challenges. For instance, environmental noise in these settings degrades alarm sounds, and multiple alarms are often issued simultaneously. The current evaluation of these factors remains insufficient, warranting further investigation.

This study aimed to develop an ML model for identifying auditory alarms issued by medical equipment. The following steps were undertaken. First, we evaluated the characteristics of old and new medical equipment alarms, as defined by IEC 60601-1-8. Next, we assessed the characteristics of auditory alarms when combined with various environmental sounds. Finally, based on the obtained findings, an ML model was constructed to identify degraded alarms and those occurring simultaneously and frequently.

## Materials and methods

Sound characteristics of old and new auditory alarms

**Figure 1:**
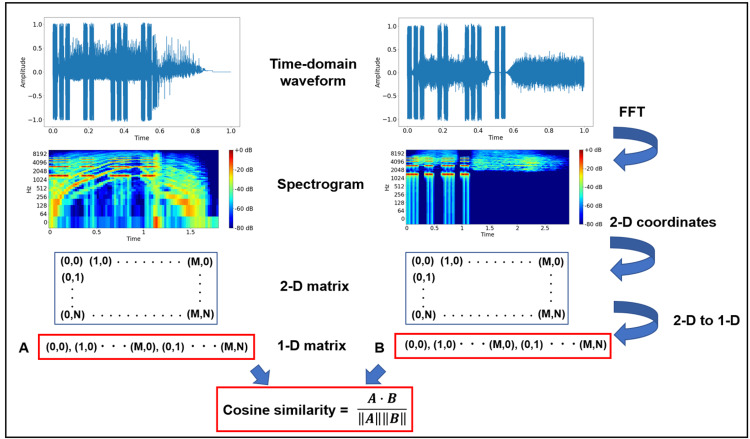
Schematic of the cosine similarity calculation for sample auditory alarms. 1-D: one dimension; 2-D, two dimensions; FFT: fast Fourier transform.

This study evaluated the frequency characteristics and similarity of auditory alarms. The analysis focused on the sample sounds of old and new medical equipment alarms outlined in IEC 60601-1-8 [9]. Notably, the IEC 60601-1-8 standard mandates that specific tones must alert personnel of equipment malfunctions, process abnormalities, and changes in patient pathology, such as power supply failure, completion of infusion, and the occurrence of arrhythmias. For example, the cardiovascular icon defined by the old standard uses trumpet call, call to arms, and major chord. However, its new standard uses "lun-dup" heartbeat sound. The previous edition of the standard stipulated that the following eight categories of alarms must be structured as five-pulse tone sequences with distinct patterns: cardiovascular, drug delivery, general, oxygen, perfusion, power failure, temperature, and ventilation (hereafter referred to as CARD, DRUG, GEN, OXY, PERF, POWER, TEMP, and VENT, respectively). The 2020 revision introduced auditory icon sounds tailored to each of the following abnormalities: cardiovascular, drug delivery, failure, oxygen, perfusion, temperature, and ventilation (hereafter referred to as CARD, DRUG, FAIL, OXY, PERF, TEMP, and VENT, respectively). Furthermore, both editions specify different alarm tones based on the priority level (medium or high). This study exclusively focused on high-priority auditory alarms. Alarm sound samples were obtained from audio files published on the web [15,16]. A schematic of the evaluation process is presented in Figure 1. To analyze the time-domain waveforms of both old and new alarms, a fast Fourier transform (FFT) was applied to generate spectrograms using Python’s Librosa library [17]. The two-dimensional coordinate space data of the generated spectrograms were subsequently transformed into one-dimensional data, from which the cosine similarity between alarms was calculated. Cosine similarity is a measure of similarity between two non-zero vectors defined in an inner product space and applied in various fields such as pattern recognition, machine learning, decision making, and image processing [18].

Evaluation of degraded auditory alarms

**Figure 2:**
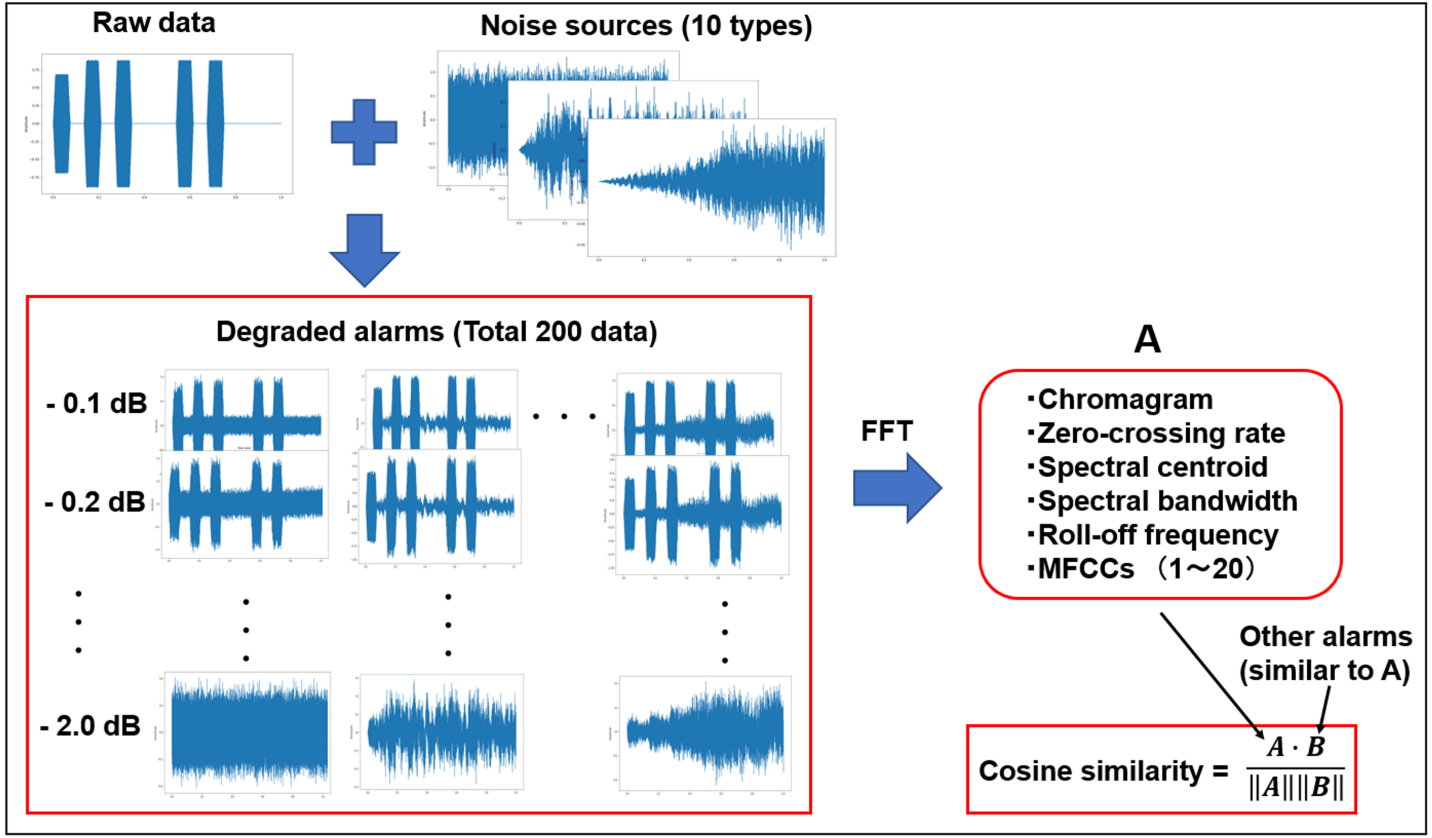
Schematic of the cosine similarity calculation for degraded alarms. FFT: fast Fourier transform; dB: decibel; MFCCs: mel-frequency cepstral coefficients.

To simulate real-world conditions, we assumed that alarms were issued in actual environments and evaluated the characteristics of alarm sounds with various superimposed noise sources. A schematic of the evaluation process is depicted in Figure 2. This analysis focused on the eight old alarm types and seven new alarm types described in the previous section. A total of 10 noise sources were used, including helicopter and aircraft engine sounds, construction noise, conversation noise, as well as white noise and pink noise. The pydub library in Python was used to synthesize sound sources and generate training data [19]. The volume of each original alarm was incrementally reduced in 0.1 dB steps, ranging from 0.1 to 2.0 dB, before being combined with each noise source. Thus, each alarm was modified across 20 volume levels and paired with one of 10 noise types, generating 200 data samples. Z-score normalization was applied to the dataset. Next, Python’s Librosa library was used to obtain key acoustic features, including the chromagram, zero-crossing rate, spectral centroid, spectral bandwidth, spectral roll-off frequency, and the first 20 mel-frequency cepstral coefficients (MFCCs) [17,20]. To evaluate the similarity between different alarm sounds under degraded conditions, we computed the average value of each index across the 200 data samples per alarm. Cosine similarity was then calculated based on these averaged feature values.

ML-based identification of auditory alarms

**Figure 3:**
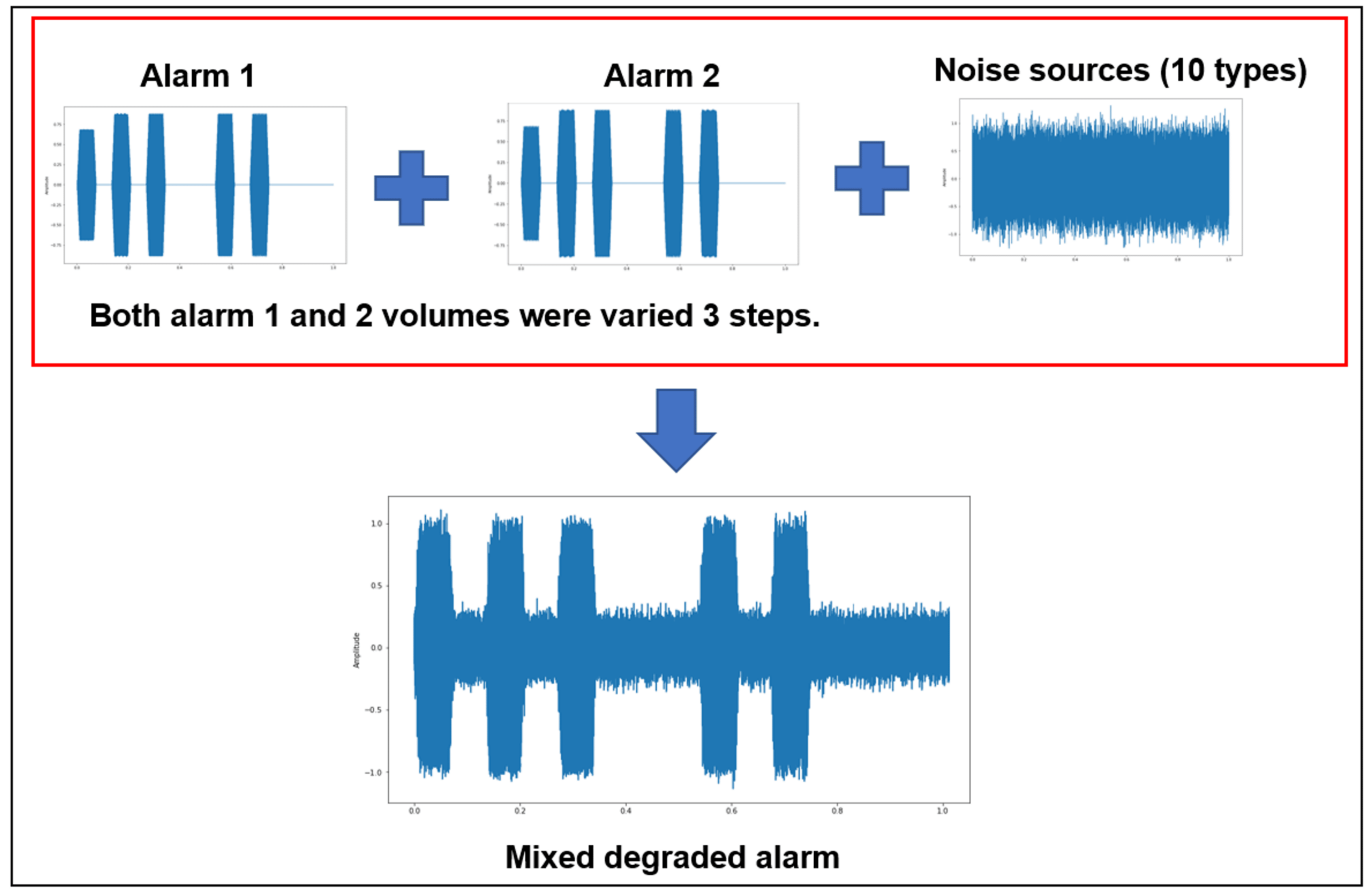
Schematic of the dataset construction process for identifying two simultaneously sounding alarms.

We evaluated the accuracy of ML-based identification of deteriorated alarm sound sources in both the old and new alarm groups. This study comprised two tasks: single-alarm identification, which involved identifying one type of degraded alarm, and multi-alarm identification, which involved identifying two overlapping alarm sound sources. The analysis targeted the eight types of old alarms and seven types of new alarms, as specified in IEC 60601-1-8. For the single-alarm identification task, we used the same dataset as that specified in the previous section, where each alarm was modified across 20 volume levels and combined with one of 10 noise types. ML models were then trained and validated using this dataset. In the multi-alarm identification task, two different alarms were first synthesized at the same volume. Figure 3 illustrates the dataset construction process. Next, a sound source containing these two alarms was combined with one noise type from the 10 noise categories. During this process, the volume of the alarm sound source was varied in three steps: 5 dB, 15 dB, and 25 dB. The final dataset was constructed for training and validation.

**Figure 4:**
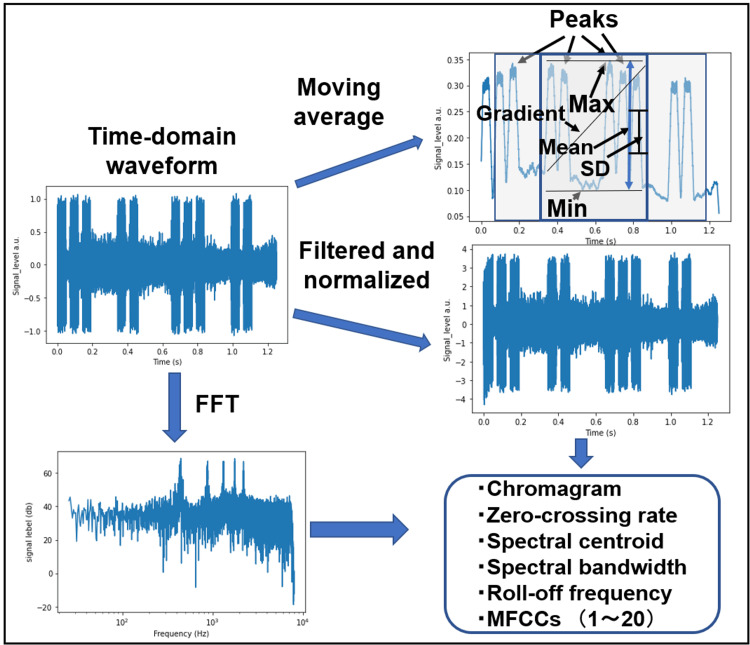
Schematic of the signal processing steps used to extract features. FFT: fast Fourier transform; Max: maximum; Min: minimum; MFCCs: mel-frequency cepstral coefficients; SD: standard deviation.

In this study, we extracted 31-dimensional features from the measured data using signal processing techniques to make the various characteristics of alarm sounds into ML [21]. Figure 4 illustrates the signal processing framework. First, simple moving averages with an interval of 300 points were applied to the time-domain waveforms to detect envelope waves and extract low-frequency features. The mean, standard deviation, minimum (Min), maximum (Max), and gradient were then computed from envelope waves extracted from the middle one-third of the total time. Additionally, the number of peaks in the envelope waves was counted within the time range spanning from one-eighth to three-fourths of the total duration. For high-frequency feature extraction, a Butterworth filter with a passband edge frequency of 10 Hz and a stopband edge frequency of 40 Hz was applied to the time-domain waveforms. To eliminate the influence of data on different scales and make model learning smoother, the filtered data underwent Z-score normalization before further processing. Next, an FFT was applied to compute the frequency spectra. Finally, spectral features, including the chromagram, zero-crossing rate, spectral centroid, spectral bandwidth, roll-off frequency, and the first 20 MFCCs, were extracted using the Librosa library.

Candidates for the regressors and classifiers included the random forest (RF) and support vector machine (SVM) algorithms. Hyperparameters were tuned for each of these classifiers, including the number of trees and maximum depth for RF, as well as the radial basis function kernel parameter (gamma) and regularization parameter (C) for SVM. Optimal hyperparameters were selected after tuning. To prevent overfitting, we employed a stratified group K-fold cross-validation method with K = 5. The model's performance was evaluated using overall accuracy. Additionally, recall, precision, and F-measure were calculated for each alarm. To assess these evaluation metrics, the data of each subject were divided into 90% training data for supervised learning and 10% test data for evaluation.

## Results

Characteristics of old and new auditory alarms

**Figure 5:**
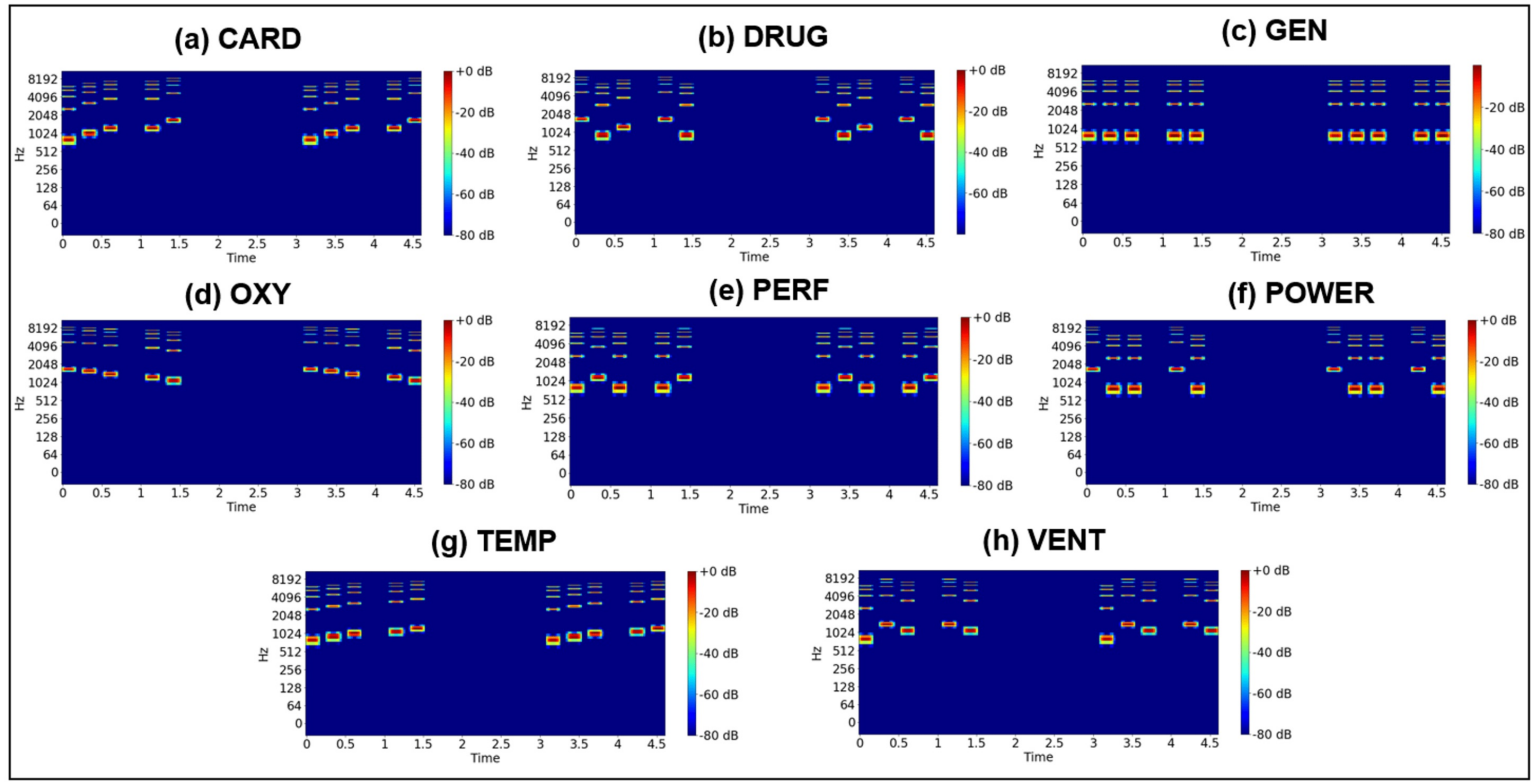
Spectrograms of auditory alarms based on the old standard. CARD: cardiovascular; DRUG: drug delivery; GEN: general; OXY: oxygen; PERF: perfusion; POWER: power failure; TEMP: temperature; VENT: ventilation.

**Figure 6:**
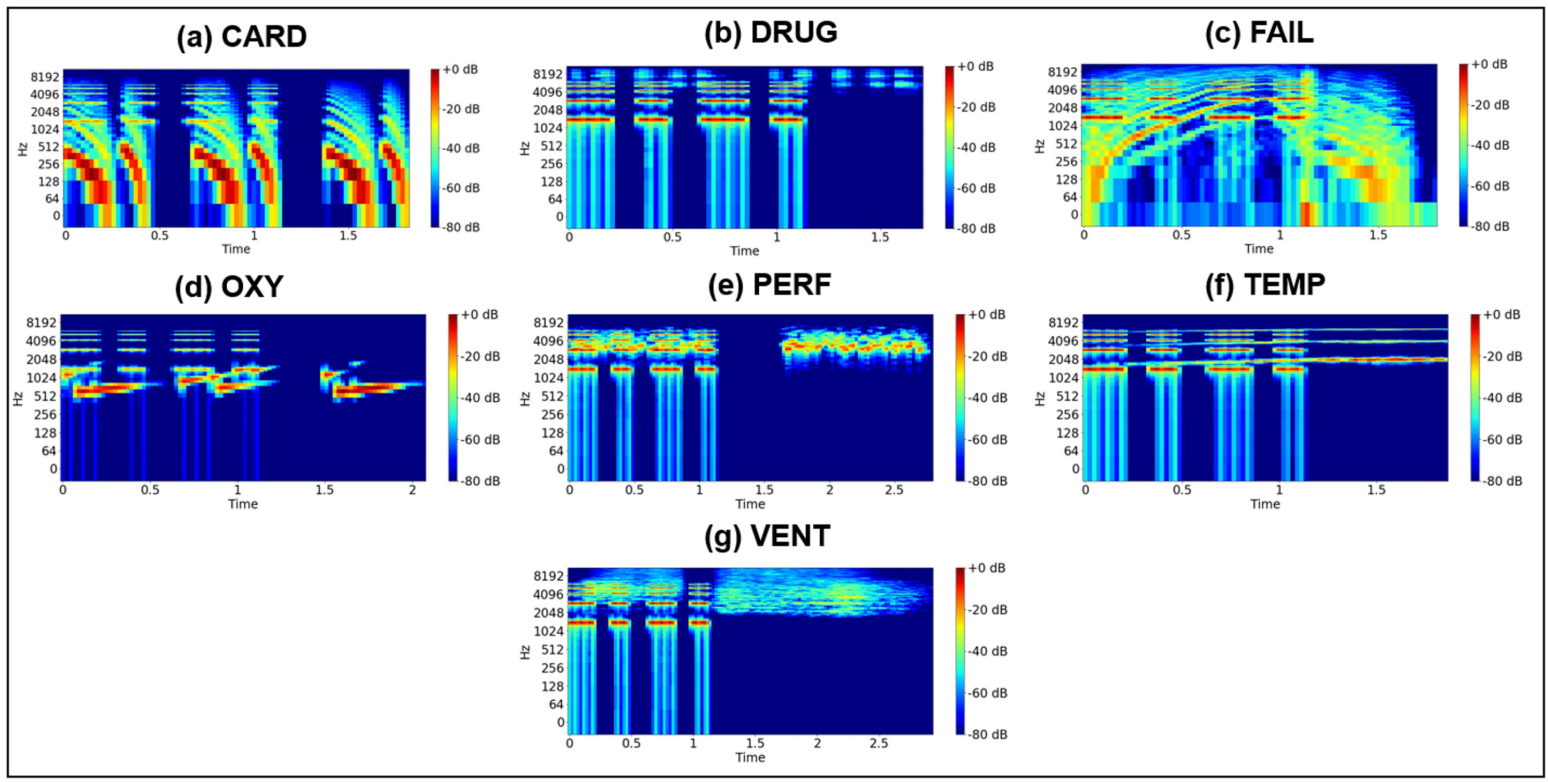
Spectrograms of auditory alarms based on the new standard. CARD: cardiovascular; DRUG: drug delivery; FAIL: failure; OXY: oxygen; PERF: perfusion; TEMP: temperature; VENT: ventilation.

Figures 5, 6 depict the spectrograms of the original high-priority alarm sounds for the eight types of old alarms and the seven types of new alarms examined in this study. Notably, the old alarms exhibit fundamental frequencies between approximately 500 and 1800 Hz, with accompanying overtone frequencies. The old high-priority alarm sound includes a beep within the 500-1000 Hz range, varying in pitch over a five-step scale. While each frequency component differs slightly, human auditory perception may struggle to distinguish between them. New alarms also display a common signal around 1500 Hz with harmonic frequencies, representing a beep tone used across all high-priority alarms. However, beyond this shared signal, the spectrograms of the new alarms exhibit significant differences from those of the old alarms.

**Figure 7:**
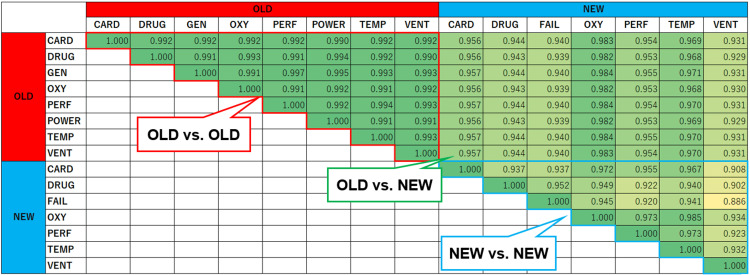
Cosine similarity values derived from the spectrogram components of old and new alarms. CARD: cardiovascular; DRUG: drug delivery; FAIL: failure; GEN: general; OXY: oxygen; PERF: perfusion; POWER: power failure; TEMP: temperature; VENT: ventilation.

Figure 7 presents the cosine similarity values computed from the spectrogram components of the old and new auditory alarms. The similarity values for all combinations of old alarms were 0.99 or higher. In contrast, the similarity values among new alarms ranged from 0.886 to 0.985. These results suggest that the new alarms are more distinguishable than the old alarms.

Evaluation of degraded auditory alarms

**Figure 8:**
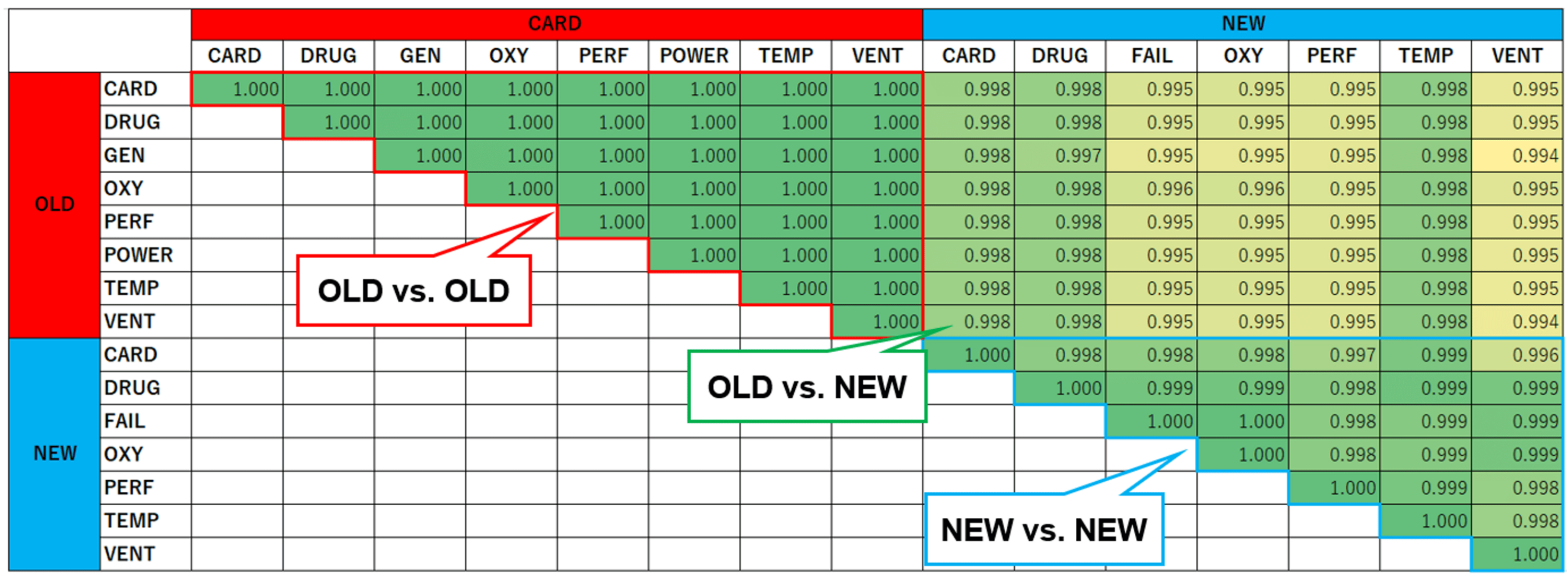
Cosine similarity values derived from the spectrogram components of degraded old and new alarms. CARD: cardiovascular; DRUG: drug delivery; FAIL: failure; GEN: general; OXY: oxygen; PERF: perfusion; POWER: power failure; TEMP: temperature; VENT: ventilation.

Figure 8 presents the cosine similarity values for degraded alarm sounds. For old alarms, all similarity values were one. In contrast, for new alarms, except for FAIL and OXY, all cosine similarity values exceeded 0.99 but remained below one. This suggests that new alarms exhibit slight spectral differences, potentially improving their distinguishability.

Estimation results of auditory alarms by ML

**Figure 9:**
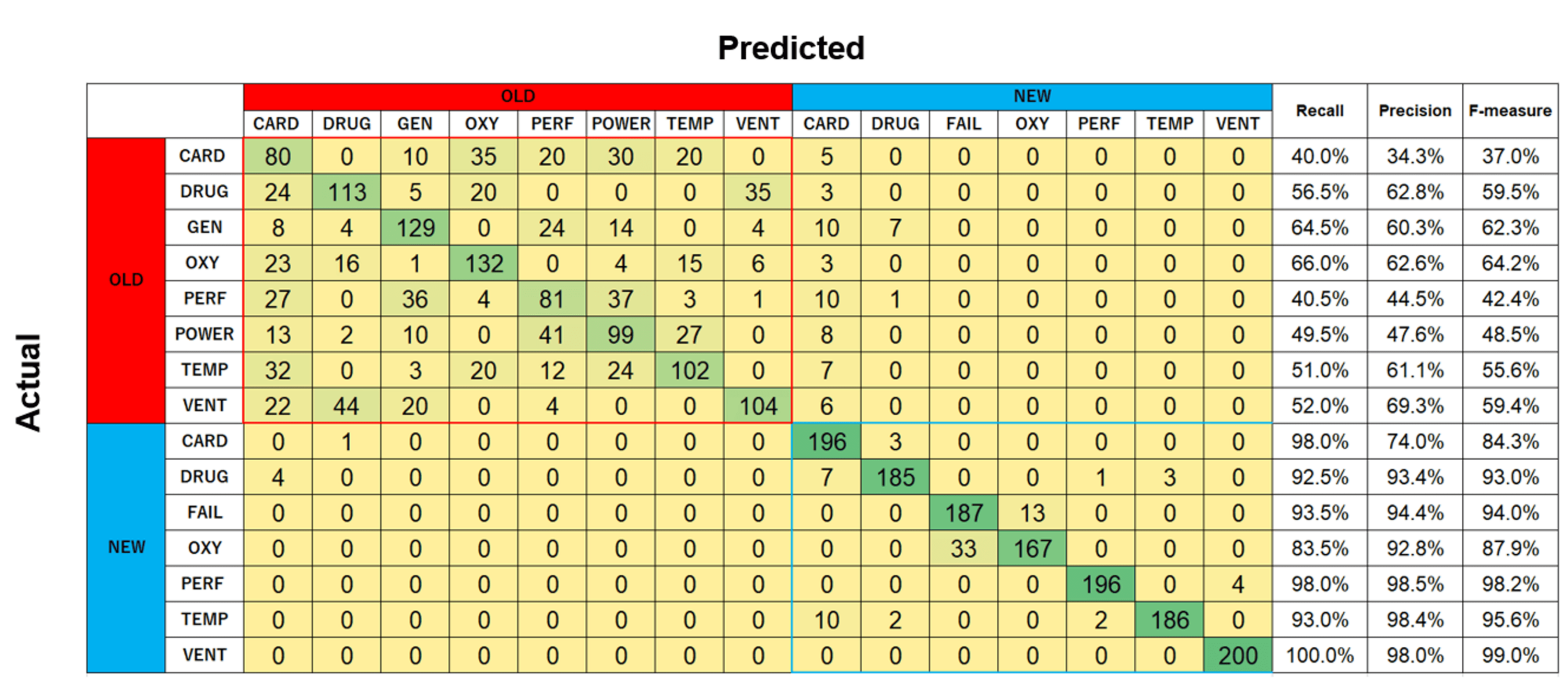
Confusion matrix for degraded old and new alarms classified using support vector machine. CARD: cardiovascular; DRUG: drug delivery; FAIL: failure; GEN: general; OXY: oxygen; PERF: perfusion; POWER: power failure; TEMP: temperature; VENT: ventilation.

The estimation accuracies for identifying a single degraded alarm sound were 64.1% and 71.9% for RF and SVM, respectively. Figure 9 presents the confusion matrix obtained by SVM for both old and new degraded alarms. The models exhibited a high number of misclassifications when identifying the old alarms, resulting in recall values ranging from 40% to 66%, precision values between 34% and 70%, and F-measure values between 37% and 64%. In contrast, the models achieved higher accuracy when classifying new alarms, with recall exceeding 80%, precision above 70%, and F-measure greater than 80% for all new alarms. Notably, VENT was identified with 100% accuracy.

**Figure 10:**
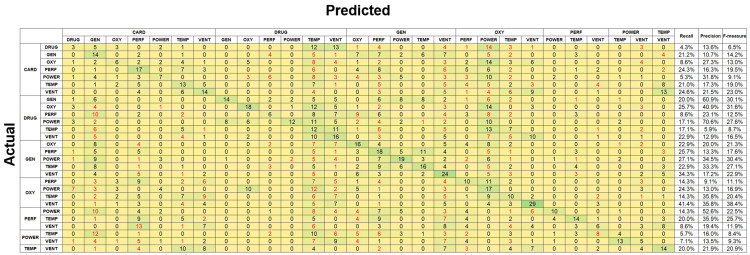
Confusion matrix for two simultaneously sounding degraded old alarms classified using support vector machine. CARD: cardiovascular; DRUG: drug delivery; FAIL: failure; GEN: general; OXY: oxygen; PERF: perfusion; POWER: power failure; TEMP: temperature; VENT: ventilation.

**Figure 11:**
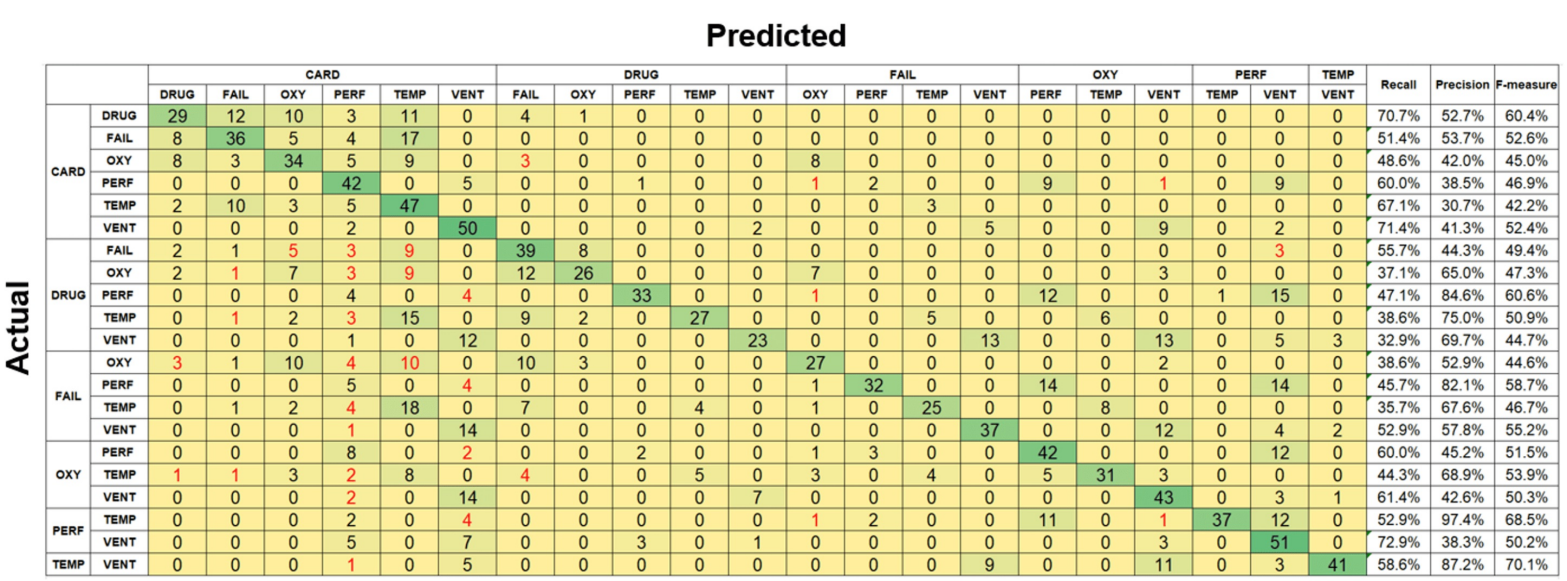
Confusion matrix for two simultaneously sounding degraded new alarms classified using random forest. CARD: cardiovascular; DRUG: drug delivery; FAIL: failure; GEN: general; OXY: oxygen; PERF: perfusion; POWER: power failure; TEMP: temperature; VENT: ventilation.

The identification accuracies for two simultaneous alarms were 15.3% and 18.6% for RF and SVM, respectively. For new alarms, RF achieved an identification accuracy of 51.2%, while SVM achieved an accuracy of 46.2%. Figure 10 presents the confusion matrix corresponding to the identification of two types of old alarms using SVM, while Figure 11 presents the confusion matrix corresponding to the identification of two types of new alarms using RF. The red numbers indicate cases where the identified alarm pair was entirely different from the correct combination. The recall rate for old alarms was low, ranging from a few percent to 30%. Although certain alarm combinations had a precision rate exceeding 70%, the F-measure remained below 40% across all cases. For new alarms, recall ranged from 30% to 70%, precision varied between 30% and 90%, and the F-measure ranged from 40% to 80%, indicating higher accuracy than that for old alarms. However, these results were still poor compared to single-alarm estimation. For instance, when only one alarm was identified, accuracy improved to 68.1% for old alarms and 94.4% for new alarms.

## Discussion

Many studies have evaluated the audibility of medical equipment alarms [11,22-24]. However, only a few have examined the audibility of alarms that are triggered simultaneously in the presence of environmental noise or attempted to identify them using ML. While some studies have explored signal processing algorithms for alarm identification, they have focused only on alarms based on the old standard, without considering techniques for distinguishing simultaneously sounding alarms [25]. Other studies have used deep learning approaches to predict simultaneously sounding alarms; however, these studies were limited to four alarm types and exclusively used old standard alarms [26]. In contrast, this study innovatively evaluates the characteristics of both old and new auditory alarms while also assessing their identification accuracy using ML.

The higher discrimination accuracy of the models for the new alarms compared to that for the old alarms is likely due to the greater tonal differences between alarms in the revised standard, as well as their distinct extracted features. This is supported by our evaluations using cosine similarity, revealing that the new alarms exhibit lower similarity between different alarm types. Additionally, even when alarm sounds are degraded by superimposed environmental sounds, the frequency components in the new alarms are designed to allow detection, unlike the pulse patterns of the old alarms. As illustrated in Figure 6, the cosine similarity of degraded alarm sounds exceeded 0.99 for new alarms owing to the influence of noise frequency components. This suggests that human auditory perception may struggle to distinguish between such degraded alarm sounds. However, ML techniques enable the detection of subtle alarm characteristics, improving identification accuracy.

Distinguishing two simultaneously sounding alarms was difficult even under the new standard. This difficulty likely arises from the following issue: as the number of simultaneously sounding alarms increases, the combined signal approaches white noise according to the central limit theorem, reducing distinctive features. Previous research has demonstrated that the identification accuracy for simultaneous alarms decreases with increasing alarm counts [26]. However, consistent with current findings, the probability of complete misidentification remains low.

In addition to the study’s findings, several limitations and future challenges also warrant discussion. This study focused on sample sounds of auditory alarms as specified in IEC 60601-1-8 rather than actual alarm sounds from medical equipment. Additionally, the evaluation used mechanically synthesized degraded sounds rather than actual speaker outputs. The study assessed the alarm discrimination accuracy of ML approaches but did not evaluate healthcare personnel’s auditory discrimination. Human auditory perception is significantly influenced by the type and volume of surrounding environmental sounds, necessitating a separate study to accurately evaluate these effects. Moreover, the same medical equipment is used multiple times in clinical settings, such as the intensive care unit. At the moment, we cannot distinguish which medical equipment is dispatching an alarm. In the future, it may be possible to identify sounds by using directional sound pickup devices and utilizing the intensity information. Another limitation is that medical equipment compliant with the new standard still includes alarms based on the old standard, meaning that both alarm types may coexist in medical settings. This study did not account for such scenarios, as it separately evaluated the distinguishability of old and new alarms. A more realistic assessment would involve evaluating a mixed model incorporating both alarm types. However, combining both old and new alarm types (15 alarms) would result in 105 possible two-alarm combinations, making it unlikely that current ML models would achieve high accuracy across all cases. In this study, we used a classical learning approach with relatively low computational cost. Future research could explore deep learning methods to improve identification accuracy.

The application of ML for identifying alarms based on the new IEC 60601-1-8 standard demonstrates that alarm sounds can be automatically detected with high accuracy. As a potential future application, detected alarm information could be integrated into the HIS and transmitted via wireless communication to mobile devices used by medical personnel. In addition to detecting alarm sounds emitted by medical equipment, this approach would enable automatic recognition of alarm signals, allowing information related to clinical condition changes or equipment malfunctions to be directly relayed to medical staff. This capability could facilitate communication limited to specific alarm types, reducing unnecessary notifications. This study focused exclusively on high-priority alarms. However, if the system could reliably distinguish high-priority alarms from moderate-level alarms, it would allow for the selective transmission of only critical alerts to medical personnel. Such improvements could help mitigate alarm fatigue. In future work, we will verify the results in an actual environment and evaluate their usefulness.

## Conclusions

This study evaluated the characteristics of old and new auditory alarms issued by medical equipment as specified in IEC 60601-1-8. The cosine similarity between old alarms was extremely high, while new alarms exhibited greater variation. When noise was superimposed, the similarity among old alarms further increased, making differentiation more challenging. In contrast, even after degradation due to noise, slight acoustic differences remained detectable in the new alarms, demonstrating their improved distinguishability. Using ML, a single-alarm under the new standard could be identified with high accuracy, even in noisy conditions. When identifying two simultaneous alarms, the overall accuracy was low; however, when at least one of the two alarms was correctly classified, the accuracy exceeded 90%.
